# A rare case of quadruple malaria infection from the highly malaria-endemic area of Bastar, Chhattisgarh, India

**DOI:** 10.1371/journal.pntd.0005558

**Published:** 2017-07-06

**Authors:** Sri Krishna, Sneha Bhandari, Praveen K. Bharti, Sanjay Basak, Neeru Singh

**Affiliations:** 1 Department of Molecular Parasitology, National Institute for Research in Tribal Health (NIRTH), Jabalpur, Madhya Pradesh, India; 2 Department of Health, District Malaria Office, Government of Chhattisgarh, Bastar, Chhattisgarh, India; Mahidol University, THAILAND

## Introduction

**Fig 1:**
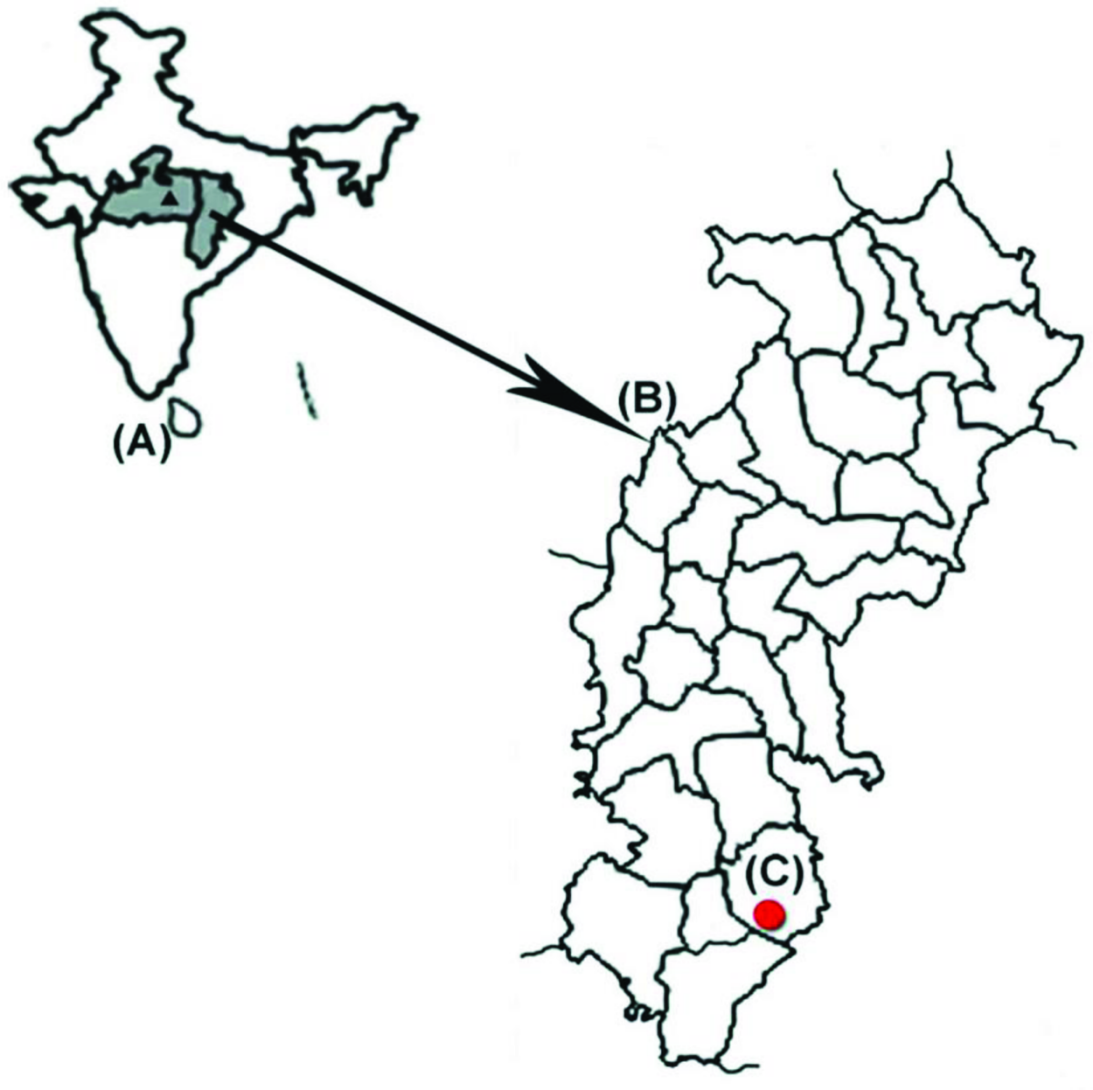
Map of India (A) showing Madhya Pradesh National Institute for Research in Tribal Health (NIRTH) and Chhattisgarh (B) and Darbha community health centre, Bastar district (C).

The past decade has seen tremendous progress in malaria control worldwide, as 57 out of 106 countries have shown a sharp reduction of about 75% in malaria incidence [1]. Despite this progress, many febrile patients are still treated with antimalarial drugs without a confirmed malaria diagnosis, because malaria diagnosis is mainly based on microscopic examination of blood smears [2, 3]. Although rapid diagnostic tests (RDTs) are easy and reliable tools for diagnosis of *Plasmodium falciparum* and *P*. *vivax*, RDTs are unable to differentiate mixed infection with uncommon parasite species, i.e., *P*. *ovale* and *P*. *malariae* [4, 5]. In this report, we are presenting a rare case having all 4 species of *Plasmodium* in peripheral blood of a young boy from a remote community health centre (CHC), Darbha, in Bastar district of Chhattisgarh state, India (Fig 1). Malaria is a major health problem in Bastar district [6]. This area is also having serious problems of insurgency, which are affecting the health services of the area adversely [7, 8]. This is the first report from India of 4 *Plasmodium* species in 1 case. Such rare cases of malaria are a diagnostic and clinical challenge.

## Diagnosis

A malaria clinic of National Institute for Research in Tribal Health (NIRTH) of the Indian Council of Medical Research (ICMR) was established at Darbha CHC of Bastar district in 2015 to provide prompt diagnosis and treatment of malaria in difficult and conflict-affected areas, as the villages in Darbha CHC are inaccessible and this region has been under continuous attack by the Maoists. There is no public transport available in the villages because of thick forest. People visit the hospital when they come to the market to purchase their daily needs.

Symptomatic patients were screened for malaria by bivalent RDT, SD Bioline Malaria Antigen P.f./P.v. (Bio Standard Diagnostics Pvt. Ltd., India), and microscopy. A finger-prick blood sample was collected from patients after taking written informed consent. Patients having doubtful parasite species were identified by molecular methods. Genomic DNA was isolated and polymerase chain reaction (PCR) was carried out for *Plasmodium* species identification using standard protocol [9]. Universal primers were designed for all 4 species of *Plasmodium*, with restriction sites for enzymes XhoI and BamHI. The full length of *18S rRNA* gene was amplified with Phusion High Fidelity DNA Polymerase (New England Biolabs, USA) and cloned into pBluescript SK (+) vector through restriction and ligation using DH5α competent cells. Amplification of the partial *18S rRNA* gene was also performed using vector-specific M13 forward primer and *Plasmodium* species-specific reverse primer from ligated product. Sequencing of *18S rRNA* from clones and ligated product was done by Di-deoxy chain termination method using 3130xl genetic analyser (Applied biosystems, USA). Genes specific to each species, *P*. *falciparum* dihydropteroate synthase gene (*Pf-dhps*), *P*. *vivax* dihydropteroate synthase gene (*Pv-dhps*), *P*. *malariae* merozoite surface protein 1 gene *(Pm-msp1)*, and *P*. *ovale* reticulocyte binding protein gene *(Po-rbp)* were also amplified and sequenced [10, 11].

## Ethical approval

This study protocol for collection of blood samples from patients with malaria infection was approved by the Institutional Ethics Committee of NIRTH, Jabalpur (Madhya Pradesh), India.

## Case presentation

A 12-year-old boy from a very remote village attended the Darbha CHC hospital with the complaint of repeated history of fever with no other complications.

At the time of admission, he gave history of 4 days’ fever and his body temperature was recorded as 100.8°F. His pulse rate was 101/min and his respiratory rate was 28/m. The laboratory findings of blood test at the time of admission were Hb≤10 gm/dL, blood glucose = 88 mg/dL, total leukocyte count (TLC) = 4,000/mm^3^, differential leukocyte count (DLC) = neutrophils (52%), lymphocyte (45%), eosinophils (2%), monocyte (1%), and serum creatinine = 0.5 mg/dL. The bivalent RDT showed the presence of *P*. *falciparum* only. Subsequently, peripheral blood smear was found positive for *P*. *falciparum* along with some doubtful structures, which a microscopist was unable to identify. He was given oral artesunate plus sulphadoxine/pyrimethamine (AS+SP). As fever was not coming down and Hb had lowered to <8 gm/dL, the patient was referred to district hospital where he was treated with intravenous antimalarial (quinine 20 mg/kg body weight on admission followed by maintenance dose of 10 mg/kg 8 hourly) along with other supportive treatment, as per the duty physician. However, he did not take the complete treatment and left hospital against medical advice. Follow-up of the patient was not possible due to inaccessibility of the area.

## Case discussion

**Fig 2:**
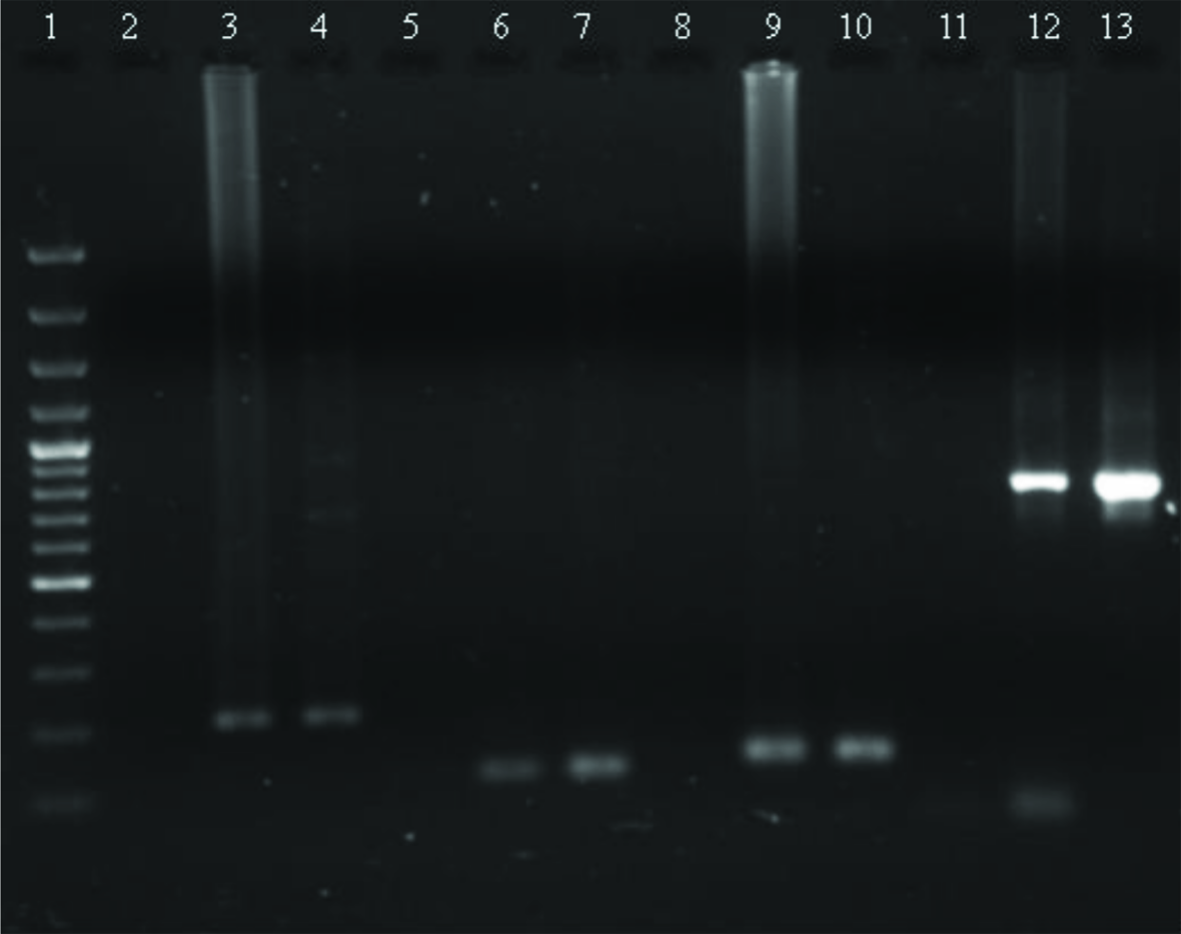
Gel image showing diagnosis of 4 *Plasmodium* species. 1: 100 base pair (bp) ladder; *P*. *falciparum* (2: NC, 3: PC, 4: sample); *P*. *vivax* (5: NC, 6: PC, 7: sample); *P*. *malariae* (8: NC, 9: PC, 10: sample); *P*. *ovale* (11: NC, 12: PC, 13: sample). Abbreviations: NC, negative control; PC, positive control.

**Fig 3:**
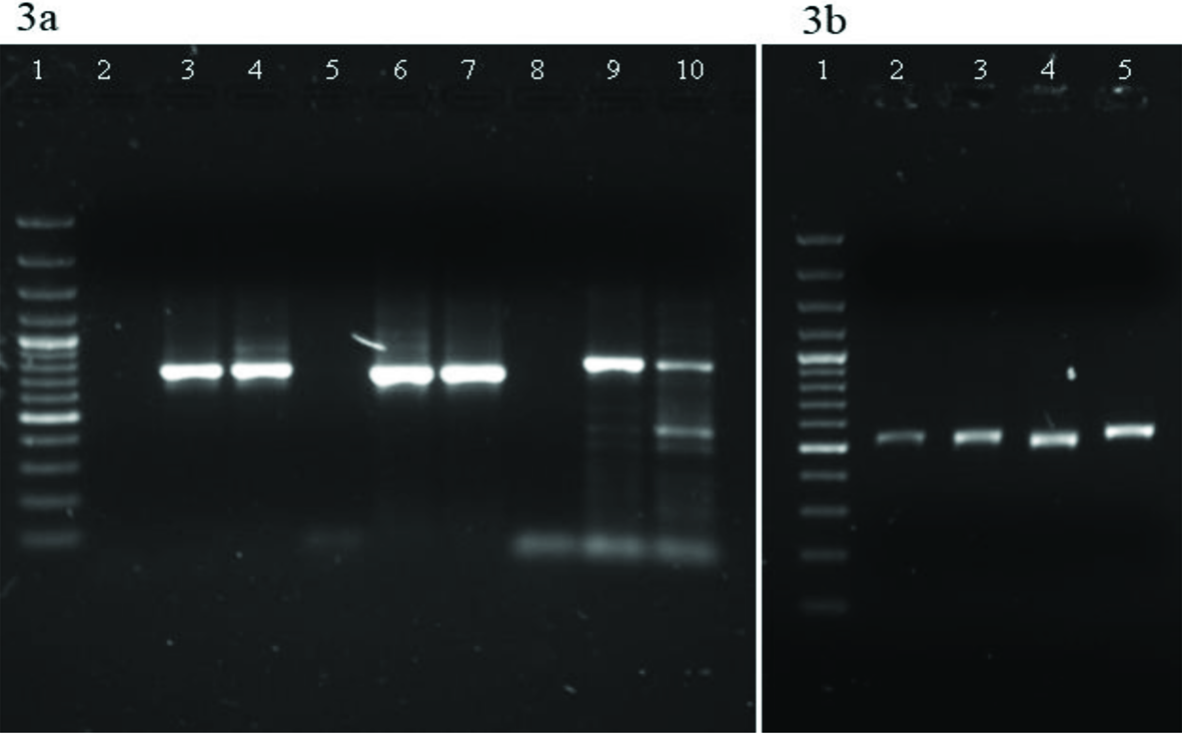
**(a) Gel image showing amplification of *Pf-dhps*, *Pv-dhps*, and *Po-rbp***. 1: 100 base pair (bp) ladder; *Pf-dhps* (2: NC, 3: PC, 4: sample); *Pv-dhps* (5: NC, 6: PC, 7: sample); *Po-rbp* (8: NC, 9: PC, 10: sample) **(b) Gel image showing amplification *of Pm-msp1* gene from genomic DNA sample**. 1: 100 bp ladder; 2–5: samples. Abbreviations: NC, negative control; PC, positive control.

In this study, 23% mixed infections were found with 2 or more species out of 160 cases of doubtful identification (Fig 2). Molecular analysis revealed that mixed infections of *P*. *vivax* and *P*. *falciparum* were highest (19%), followed by *P*. *falciparum*, *P*. *vivax*, and *P*. *malariae* (2.5%); *P*. *falciparum* and *P*. *malariae* (1.3%); and only 1 case with all 4 species (Fig 3a & 3b). All these mixed infections were mild and did not show any complications. The analysed sequences of 4 species were submitted to the GenBank database (accession numbers KU510226-KU510234 and KY202757-KY202761). Of the 4 species, *P*. *falciparum* causes the most severe symptoms, i.e., severe anaemia, cerebral malaria, multiorgan failure, and death [6, 12]. *P*. *vivax* and *P*. *ovale*, although responsible for mild infection, may persist within the liver as hypnozoites, causing relapses even after treatment with blood schizonticides [13]. *P*. *malariae* is also mild, may persist in the human population at very low density, and may cause renal failure [14]. The available results from various studies are contradictory. The studies carried out in Ivory Coast [15], Sri Lanka, [16], Thailand [17], and Vanuatu [18] revealed that in mixed-species infection, the severity may be modulated. On the contrary, others reported that mixed infection of *P*. *falciparum* and *P*. *vivax* affected the clinical outcome in patients [19–21]. It is well known that the frequencies of less common species such as *P*. *malariae* and *P*. *ovale* are largely underestimated by microscopy [4, 22]. The low sensitivity of microscopy has 2 major consequences in malaria-control efforts. First, low-density parasitemia may serve as a reservoir for infections; second, in mixed infections, the tendency of 1 parasite to dominate the other lowers the efficiency of microscopic detection of 2 species in the same sample [23]. In this study, initial inappropriate treatment was given due to incorrect diagnosis of parasite species, as it is known that SP efficacy against vivax malaria is imperfect. Incorrect malaria diagnosis is a severe public health concern, as misidentification of malaria parasites could lengthen the parasite-clearance time and lead to recrudescence [19] and drug resistance [24]. This study indicates the need of adequate training of health staff involved in malaria diagnosis and the inherent limitations of microscopic diagnosis, which is prerequisite for malaria elimination.

**Key learning points**

- A case of mixed infection of 4 *Plasmodium* species was found, which is very rare, and this is the first report of such a case in India.
- Microscopy is the gold standard for malaria diagnosis, but in case of mixed infection, 1 or more species may be missed, especially in case of low parasitemia.
- The study showed high proportion of mixed infection by molecular methods.
- High proportion of mixed infections signifies the need of adequate training of health staff involved in malaria diagnosis, which is prerequisite for malaria elimination.
